# Decolonization in health professions education: reflections on teaching through a transgressive pedagogy

**Published:** 2016-12-05

**Authors:** Ruth Rodney

**Affiliations:** 1 University of Toronto

## Abstract

**Background:**

Canadian health educators travel to the global south to provide expertise in health education. Considering the history of relations between the north and south, educators and healthcare providers from Canada should critically examine their practices and consider non-colonizing ways to relate to their Southern colleagues.

**Methods:**

Using her experience as a teacher with the Toronto Addis Ababa Academic Collaboration in Nursing, the author explored issues of identity and representation as a registered nurse and PhD candidate teaching in Ethiopia. Transgressive pedagogy was used to question how her personal, professional, and institutional identities impacted her role as a teacher.

**Results:**

Thinking and acting transgressively can decrease colonizing relations by acknowledging boundaries and limitations within present ideas of teaching and global health work and help moving beyond them. The act of being transgressive begins with a deeper understanding and consciousness of who we are as people and as educators.

**Conclusion:**

Working responsibly in the global south means being critical about historical relations and transparent about one’s own history and desires for teaching abroad.

## Introduction

As a registered nurse who has travelled globally on voluntary medical trips and now as a doctoral candidate in the roles of teacher and researcher, I am regularly questioning what it means to live and teach in non-colonizing ways. I have realized this is not an easy feat for several reasons. However, it is necessary as I work with and in a healthcare system that historically has contributed to western colonization. This very system continues to perpetuate an unequal power dynamic between the global north and south in teaching and health professions education.

As Sefa Dei stated, “Colonial refers not simply to north/south, but also to the way in which knowledge can be imposed on others through imperial relations. It is also a discursive approach to interrogate why, within communities, some knowledge is validated or privileged as opposed to other forms of knowledge.”^1^^p.70^

In this paper, I explore ways of breaking free from the colonial relations that are present in health professions education, learned through my dual experiences of being a PhD candidate and a teacher with the Toronto Addis Ababa Academic Collaboration - Nursing (TAAAC- Nursing).

I make no qualms in clearly articulating that I situate myself as a teacher and researcher within and through critical theories. This is a conscious decision. To think through a critical theoretical lens means that I do not accept society as is, but rather, I question how and why society is the way it is, how I am positioned in society, how I see myself, and also what needs to change in order to eliminate inequities for marginalized populations.^2^–^4^

In the following pages, I will begin with questioning my own positionality as a starting point and key element of engaging with decolonizing thought. I am not separate or distinct from the very systems that I critique. Thus, challenging my own history is important to continuously uproot dominant ideologies of normalcy that are based on “…highly unequal societies in which economic inequity, racism, and class discrimination are empirical realities.”^5^^p.134^ I will address my experience as a Canadian nurse and PhD candidate assuming a teacher role in a Masters program in Ethiopia. With this dynamic, there are inevitable implicit colonial relations at play. I offer some reflections on how I have used a transgressive pedagogy to challenge my thinking and practice in dismantling unequal power dynamics; this I consider necessary in decolonizing my practice as a teacher in health professions education.

### Who is Ruth Rodney? Positionality of a teacher

The answer to this question continues to evolve as life experiences reveal parts of myself that I am still learning and compels me to look back and reconsider what I thought I knew. At this point in my life, who I am, where I position myself, and how the world positions me reflects all that is deeply complicated, unfixed, and yet exhilarating about this world. To know myself is to know my history, which I have come to realize is one of the main reasons I choose to work in the Caribbean, South America, and Africa as part of my life’s work. My name symbolizes a narrative of the complete erasure of part of my history, and to me, is one of the most personal and powerful ways to strip someone of their culture. So, when I travel to Guyana or to other countries, irrespective of the professional role I am in, it is always a deeply personal experience because in many respects it is helping me to explore my own identity. I am a black Canadian woman of Guyanese heritage who is a wife, sister, aunt, activist, nurse, new researcher, and teacher.

Growing up as a second generation black Canadian in Southwestern Ontario, I was constantly asked where I was from. When I would say that I was Canadian, the follow up question was always, “No, no, where are you *really* from?” as if being black could not and would not be Canadian. I would respond to this question stating, “Guyana,” as I am the youngest in my family and the only person Canadian born. Identifying as Guyanese became a major part of my identity growing up, because I did not really know or feel what it meant to be Canadian. Today, I would identify myself as Canadian of Guyanese heritage, recognizing that what I had experienced for so many years growing up was a form of “othering.”

As critical theorists Said and Fanon have explained, othering occurs when people are defined by how different they are in relation to a European idea of humanness.^6^,^7^ By this, I mean that in my own life there was a whiteness that represented what it meant to be Canadian and my blackness was the “other” to that definition. Being reflexive, I realize I also “othered” others, often asking where they were from if they were racialized. This is problematic in two ways: first, I reproduced the same racism I experienced, and secondly, I made an assumption that white skin represented a collective identity.^8^,^9^

As a nurse, I have worked globally in clinical roles over the past 12 years enhancing my practice through experiential learning.^10^ Kolb’s theory suggests that experiences can be used to transform how learners think about various healthcare practices or health issues through different learning styles.^10^

One of the most influential trips that challenged me to re-think how I conceptualized public health, healthcare, and my role took place in 2006 when I volunteered at Matangwe Clinic in rural Kenya, for Caring Partners Global.^11^ During this trip, I gained a better understanding that health was not just defined in a clinical capacity, but also through the social determinants of health.

When I returned home, I was very introspective. I started questioning how much I truly helped the patients I served. While I provided medical care on a daily basis, I knew that my contribution to patients’ health, though perhaps immediately valuable, had little effect in changing the living conditions that gave rise to many of their health problems. This experience solidified my desire to expand my nursing practice into more sustainable ways of helping communities.

In 2015, I ran into the founders of Caring Partners Global, Mr. Stephen Scott and Mrs. Sylvia Scott, who live in Canada, but are Kenyan born and from Matangwe. They informed me that the clinic has now developed into a hospital. The hospital designation provides access to financial resources from the Ministry of Health and non-governmental organizations (NGOs) to expand their services and provide more care to the community.^11^ Looking back now on that trip and the progress that has been achieved since then, I realize that it provided an ideal example to learn what vision, dedication, and working with communities in meaningful ways can achieve.

**Figure 1:**
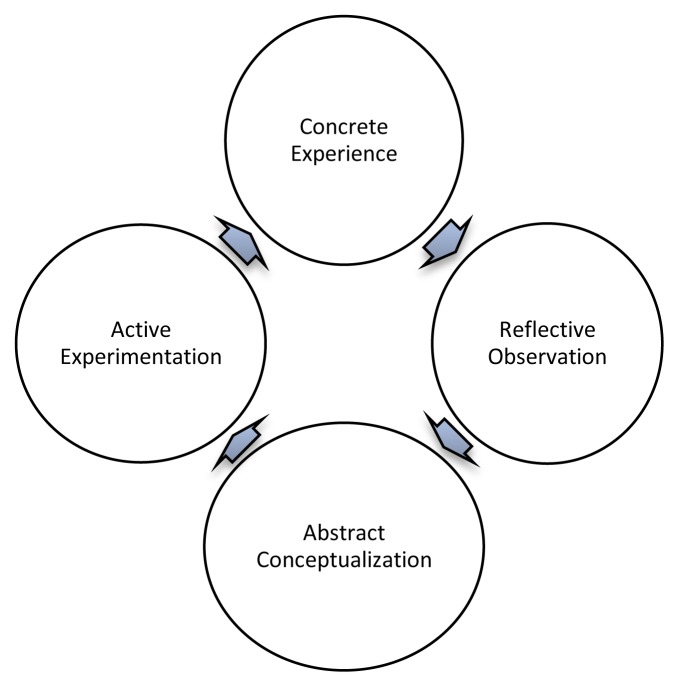
Kolb’s Learning Cycle

Considering Kolb’s learning cycle (Figure 1), I have transitioned through each stage.^10^ Now, as a doctoral candidate and new teacher, I am once again using active experience to engage critically and learn through my experiences.

### Experience of a Canadian nurse teaching in Ethiopia

TAAAC-Nursing is a part of the Toronto Addis Ababa Academic Collaboration (TAAAC), a university wide partnership between University of Toronto (U of T) and Addis Ababa University (AAU).^12^,^13^ The specific collaboration between U of T’s Bloomberg Faculty of Nursing and AAU’s Department of Nursing has been focused on the development of Ethiopian academic nursing leadership through the Masters of Nursing program at AAU.^12^ With the recognition of unequal power dynamics that often impact working relationships between north-south partnerships, the priorities of TAAAC-Nursing activities are always directed by the Ethiopian partners who determine what is needed from their Canadian colleagues.^12^

I initially learned about TAAAC-Nursing in 2013 and my first step was a conversation with Dr. Amy Bender, the TAAAC-Nursing program coordinator. All of my previous experience had taught me to question north-south partnerships such as this, specifically to wonder about the motivations and purpose of organizations of the north working “abroad.” This conversation with Dr. Bender clarified for me that we shared a worldview that rested on social justice as the underlying value for all global health work. Social justice to us means working towards eliminating systemic barriers globally that negatively impact the social determinants of health. Together we recognized that I would be a good fit for the program and the program was a good fit for me.

Prior to travelling I had mixed emotions of excitement and confusion. I was excited to be able to have the opportunity to once again set foot on a continent that holds the mystery of my ancestry – an ancestry that in many ways has always remained unknown to me. As Sefa Dei has indicated, “it is generally accepted that the influence of globalization is far-reaching. Scripting human lives, bodies, and communities, globalization has found its way in every facet of the African existence – social, material, physical, spiritual, cultural, political, economic, and psychological.”^14^^p.68^ Therefore, Ethiopia has inevitably been influenced by globalization.

Italy’s attempts to colonize Ethiopia have created debates on whether the country was colonized in the same way as other African nations during the same time period.^15^ Notably, the treaty of Wichale in the nineteenth century created a contentious relationship with each country claiming power and authority for their country’s foreign relations.^15^ Italy’s eventual acknowledgement of Ethiopia as an independent state, through the treaty of Addis Ababa and the defeat of Italy’s army by Ethiopia, was viewed as a victory in resisting colonization by Ethiopia and other African nations.^15^ Therefore, for the soles of my feet to walk upon a land that had not been colonized rejuvenated my soul in many ways.

However, I also felt confused. I had to resolve my preconceived ideas about the role of a teacher and how this was further complicated by teaching “medical” knowledge globally. Our role, as the Canadian partners, was to share knowledge, resources, and critical care expertise from our context with nursing students in the Ethiopian context. With critical care being a new area of specialization for the country, this seemed a valuable contribution to be making.

Nevertheless, the question then becomes, how do we teach without colonizing (i.e. setting ourselves as the model, establishing our values as superior) our Ethiopian colleagues when we operate from Western biomedical ideas of nursing and knowledge production? This was difficult for me to resolve. In one sense, a body is a body anywhere in the world, and trauma and acute disease damage the physical body. Although context may change how the body is handled and diseases are managed, there is great value in understanding it in this concrete biomedical way.

In another sense, I operate from a set of guidelines that are regulated by the College of Nurses of Ontario. I was also trained by an incredibly strict nurse preceptor in my undergraduate degree. She taught me a very high standard of clinical practice that I still adhere to, and I realized that my thinking around the practice of nursing and how it should be taught comes from this experience. This impacts how and what I teach other nurses, and also what I as a teacher expect from students.

Additionally, as a racialized Canadian, who has often been positioned in the margins of society, it was uncomfortable to realize that I am respected as an expert, simply by virtue of where I live and the historical relations between the north and south. I wrestled with how my teaching, and even the program as a whole, could perpetuate the decentering of Africa and African ways of knowing in relation to the global north. To continuously “check-in” with myself to ensure I was always aware of the unavoidable power dynamics between the Ethiopian colleagues and myself, I chose to approach and reflect on my teaching through a transgressive pedagogy.

### Transgressive pedagogy

Hooks (*sic*), a feminist theorist, activist, writer, cultural critic, and professor, defines a transgressive pedagogy as one that expands beyond societal and institutional boundaries by engaging directly with questions of bias that perpetuate systems of domination, and also finds new ways to teach diverse groups of students.^16^ To me, being transgressive is not simply a role you put on when you step into a classroom as a teacher or when you enter institutions or settings that represent unequal power dynamics.^16^ To believe that you only focus on being transgressive when standing in the front of a classroom means that a movement against and beyond the defined boundaries of teaching is not particularly conceivable because you have already been limited by the definitions of that role. To be transgressive is to think and live transgressively every day.

### Personal transgressive-ness

In their discussion on aboriginal research methodology, Absolon and Willet highlight the importance of the researcher’s positionality to Indigenous methodologies.^17^ They indicate that “neutrality or objectivity do not exist in research, since all research is conducted and observed through human epistemological lenses.”^17^^p.97^ Therefore, it is necessary for researchers who will contribute to new knowledge to explicitly locate themselves for transparency and self-accountability.^17^ Although Absolon and Willet are addressing researchers, these principles can be applied to teaching.^17^

It is not possible to teach from a place of neutrality or objectivity. My positionality as a teacher necessarily influences what and how I teach. Values, biases, previous learning, and experiences all have an impact on how I understand and how I approach my relationship with students, as well the students themselves. In fact, to believe that a teacher can teach from an objective, detached viewpoint is related to the reproduction of the status quo, and thus operates in colonizing ways. A teacher may believe that they are challenging inequalities by providing education to those who have been deprived of that particular knowledge. However, without a clear understanding of how we are perceived as teachers and the privileges we take for granted, we can continue to perpetuate colonizing relations. If, as Sefa Dei states, that “decolonization questions the paradigms through which we view ourselves individually/collectively, our place in the world, and our vision of that world past, present and future,” a decolonized teacher has to problematize her beliefs and practices.^14^^p.61^

### Educational transgressions

“I have been working to change the way I speak and write, to incorporate in the manner of telling a sense of place, of not just who I am in the present but where I am coming from, the multiple voices within me. I have confronted silence, inarticulateness. When I say, then, that these words emerge from suffering, I refer to that personal struggle to name that location from which I come to voice – that space of my theorizing”.^2^^p.146^

During these past four years I have travelled to Guyana several times as my doctoral research is taking place within the capital city of Georgetown. On one of my earlier trips, my aunt and I visited Pandama, a serene retreat and winery located in the lush greenery of Guyana. While there, we took a walk with one of the owners (Tracy) to a small stream that runs through the property. My aunt took a glass, dipped it into the stream, and drank the water. She then handed me the glass and told me to drink the water from the stream. As I looked down at the tea coloured water with tree leaves floating in it, I politely said, “no thank you.” Tracy and my aunt kept insisting that I drink the water, to which I kept replying, “no thank you, because, I would get sick.” Tracy then said, “sometimes we have to give ourselves permission to do things we are told we cannot. You have been programmed to believe that this water is not good. You will not get sick.” I then decided to drink the water and I never got sick.

I would describe my present location as a PhD candidate like this event at the stream. When I entered the program, I had particular beliefs and opinions that I expressed as ultimate truths. These truths were based on past education and teachings I had learned as a youth. While I believed that I was open-minded, I now know that I was mainly regurgitating what I had been taught within a traditional, uncritical system. I definitely had questioned what I was taught throughout my life, (which I believe inevitably directed me towards my PhD), but I did not have all of the tools to fully understand how deep this colonization process was ingrained in me.

These past four years have been a process of decolonization because I released many opinions that I held as an ultimate truth and have continuously re-visited who I am, who and what I represent from the past, present, and for the future. Drinking the water at the stream that day was a symbolic step towards dismantling all that has been taught to me and “giving myself permission” to create my own knowledge, re-learn more about my own history, and recognize that knowledge and ways of knowing come from many different places and in diverse forms. “Seeking our truth in our location aids us in recovering ourselves and our strengths, and in uncovering historical oppressions. Our perceptions of who we are and how we locate ourselves are a result of our own personal and political consciousness. Nonetheless, recovering truth inherently implies taking off the blinders to become conscious.”^17^^p.119^ Centering myself throughout these four years and unlearning much of what I had been taught has provided me with a deeper understanding of the impact that education and teaching has on people in their personal and professional roles. However, I must also recognize that having allocated time to simply think and develop my thoughts, together with opportunities to converse with different thinkers would not have been accessible had I not been in this program, which is a privileged position.

### Professional transgressions

Like myself, I am sure that many other nurses have been asked why they did not become a doctor or been told that to improve as a nurse means to become a physician. These comments reflect a longstanding history of a professional hierarchy within the healthcare system, where nurses have been steadfast in challenging limited perceptions of a nurse’s role and redefining the spaces that we can occupy.^18^ Essentially, the profession of nursing has frequently operated at the periphery of power and prestige in healthcare systems. Even though I would argue that nurses are the backbone of this system, our skills are not always valued.^18^

When I thought about working in Ethiopia, I was focused primarily on the individual interactions between my fellow travel teacher, the Ethiopian nurses, and myself. However, I did not consider the surrounding space or the dynamics between physicians and nurses that could impact our teaching environment. On this trip, the pursuit of greater knowledge proved to be a reminder of the realities our profession faces when attempting to expand the role of nurses.

During my visit, the Ethiopian nurses were told they could not practice clinical skills within the intensive care unit unless it was performing traditional nursing tasks such as bathing patients. They were also told that the education of medical students took precedence over their need to learn and therefore, they could not be in the intensive care unit at all. The complexities of the situation were made even more evident as staff nurses understood the unfairness of the situation but did not feel they were in a position of power to challenge it.

While we were requesting students not to engage those physicians who were perturbed by the presence of our group, these students stood strong in their desire to learn. Rectifying this situation did not happen without help from the organizers of TAAAC and TAAAC-Nursing, as the tension was most likely caused by our approach as foreign teachers who did not know how to effectively prevent or address conflict in working relationships within this local context.

Initially, I thought about this situation in relation to my role as an emergency room nurse where the working relationships between most physicians and nurses are quite good. Through this perspective, it would be easy to see this as a situation that occurs in healthcare systems “over there,” where the nursing profession is at a different place in their professional spectrum. However, through a critical perspective, I acknowledge that the department I currently work in also faces similar challenges with the sharing of learning opportunities for resident doctors and nursing students.

As I reflect back on that situation, I recognize a missed opportunity to engage in further conversations with Ethiopian nurses about how they navigate tensions such as the ones experienced in the ICU. Being in the country for less than one month when this situation occurred, I was not quick to challenge or move beyond established boundaries because I did not have a complete understanding of the dynamics within the local healthcare system. In this situation, I chose to remain in the traditional role of a teacher and let the program coordinator, Vanessa Wright, address the situation by engaging in conversations with departmental heads and the organizers of both TAAAC and TAAAC-Nursing. These conversations were necessary to understand and learn how our Ethiopian physician colleagues interpreted our presence and approach as disrespectful.

However, thinking through the entire teaching experience I believe that to contribute to Ethiopia’s nursing profession, in the manner that TAAAC-Nursing has developed, is a form of resistance. The difference in this program is that Canadian nurses contributed to providing expertise for nurses to operate in leadership positions as educators. This in itself is transgressive because as Ethiopian nurses gain more expertise and different skill sets, they can possibly provide opportunities for other Ethiopian nurses to branch out into different settings. So, teaching was about technical knowledge, but also ways of collaborating, valuing difference, and creating strategies for survival.

Negotiating the possibility of transgressiveness also meant recognizing the potential of colonizing practices, as this experience has shown. To deal with this reality, I was aware of my actions and more importantly, I paid keen attention to how the Ethiopian colleagues responded to my actions. For me, the best indicators that I was transgressive came from my careful observation and the communication with some nurses once this trip was finished.

### Institutional transgressions

“It [Academia] is that contradictory place where knowledge is colonized but also contested – a place that engenders student mobilizations and progressive movements of various kinds. It is one of the few remaining spaces in a rapidly privatized world that offers some semblance of a public arena for dialogue, engagement, and visioning of democracy and justice. Although these spaces are shrinking rapidly, dialogue, disagreement, and controversy are still possible and sanctioned in the academy”.^3^^p.170^

I have chosen to consider the university as an institution for transgressiveness for several reasons. First, it is a space that I have occupied for the past four years as a student. As hooks stated, I have felt both the pressures to conform to a particular way of speaking and writing, yet also challenged in many ways to question my own thinking.^2^ Secondly, it is through this space that I had the opportunity to teach nurses in Ethiopia. Lastly, the discussion on transgressive pedagogy in teaching would not be complete without considering the space in which one needs to be and chooses to be transgressive.

The main question I considered was, to what extent does this institution (University of Toronto, Faculty of Nursing) provide spaces for engagement and practice in global health work and teaching, that “challenge[s] the politics of knowledge that naturalizes global capitalism and business-as-usual in North American higher education?”^3^^p.171^

As a student within the academic institution, I was encouraged by my supervisor, Dr. Denise Gastaldo, to participate in this trip. Working under a supervisor who is not focused on reproducing academics that replicate her own work, but rather, chooses to encourage students to pursue their own passions and interests is in itself a form of resistance. However, the dominant system of academia does not support this way of teaching students as Mohanty has stated.^3^

Furthermore, the opportunities and time provided within the PhD program to learn critical theories and qualitative research methodologies and methods is important as a new teacher. It has taught me to be more accountable and conscious about the decisions I make as an educator and knowledge creator.

I cannot speak to the process the organizers of TAAAC-Nursing had to overcome to implement this program; however, I consider the words of Dr. Robert Hill who spoke to students earlier this year in a lecture I attended titled, “And still we rise: A new generation of black students arises for a new time.”

He encouraged us to ask the question - what time is it? From this lecture, I understood that our movements beyond and against boundaries are always on a continuum of time and therefore, we have to know where we are entering into history. Considering his words, I can surmise that the development of TAAAC-Nursing is also dependent on where this program enters into the institutional goals and mandate of both U of T and AAU. Leadership, dedication, and shared values between both sites would be the driving force for the continuation and expansion of this program.

This discussion on institutional transgressions is by no means exhaustive, as I believe that there are complexities in how transgressive spaces are occupied and accessed within large institutions on different levels. For example, I am focusing on understanding spaces where the Faculty of Nursing provides opportunities to move beyond and against boundaries; however, the Faculty of Nursing must also be considered within a larger university community that has its own hierarchies of academic knowledge, power dynamics, and financial obligations. This I also consider with respect to our Ethiopian partners and wonder how similar their need is to be transgressive within their own environment.

### Conclusion

Globalization has provided opportunities for us as Canadian educators to teach and learn abroad, but we must be aware of the implications of north-south partnerships and of education being used to colonize our colleagues in the global south. It is inevitable that we will continue to travel, work, and teach globally but this must be done in a responsible way with an acknowledgement of who we are, the privileges we are afforded, and what our perspectives are of who we interact with when we teach. To be aware means to acknowledge that we are constantly a work in progress and that every day provides new opportunities to be reflective and critical in how we operate in this world especially as educators. This process is not easy, and we will face struggles and uneasiness in acknowledging our own shortcomings. However, this is a necessary process in the pursuit of social justice. Applying a transgressive pedagogy to my teaching experience provided an opportunity for me to think more critically about the spaces I occupy in my teaching and how I can continue to move against and beyond set boundaries. I believe that this process begins with a better understanding of self, which is the basis from which professional and institutional identities are built.
